# Cross Cultural Workers for women and families from migrant and refugee backgrounds: a mixed-methods study of service providers perceptions

**DOI:** 10.1186/s12905-021-01368-4

**Published:** 2021-05-27

**Authors:** Helen J. Rogers, Lily Hogan, Dominiek Coates, Caroline S. E. Homer, Amanda Henry

**Affiliations:** 1 Child, Youth and Family Services, South Eastern Sydney Local Health District, Sydney, NSW 2010 Australia; 2 School of Women’s and Children’s Health, University of NSW (UNSW), Sydney, NSW 2052 Australia; 3 Centre for Midwifery and Child and Family Health, Faculty of Health, University of Technology Sydney, Sydney, NSW 2007 Australia; 4 Maternal and Child Health, Burnet Institute, Melbourne, VIC 3004 Australia; 5 Department of Women’s and Children’s Health, St George Hospital, Sydney, NSW 2217 Australia; 6 Australia Global Women’s Health Program, The George Institute for Global Health, Sydney, NSW 2042 Australia

**Keywords:** Migrant, Refugee, Pregnancy, Postnatal, Bilingual workers, Service providers, Culturally responsive

## Abstract

**Background:**

Women from migrant and refugee backgrounds who live in high-income countries are at increased risk of adverse perinatal outcomes, including mental health issues, preterm birth and maternal and infant mortality. There is a need to implement and evaluate models of care to meet their specific needs in order to improve health outcomes, their experiences of care, and overcome barriers to access. In Sydney, Australia, a unique model of care was implemented to support women and families from migrant and refugee backgrounds to access health and community-based services through the continuum of pregnancy to the early parenting period. This model of care is known as the Cross Cultural Workers (CCWs) in Maternity and Child and Family Health Service (the CCW Service). The aim of this study was to explore the perceptions of service providers regarding the CCW Service and identify recommendations for improvement.

**Methods:**

A mixed-methods study was conducted consisting of surveys and face to face semi-structured interviews. Service providers were recruited from hospital-based maternity and community-based services. Survey data were analysed descriptively. Interviews were analysed using qualitative content analysis.

**Results:**

Sixty-nine service providers completed surveys and 19 were interviewed. The CCW Service was highly regarded by service providers who perceived it to be critical in improving care for women from migrant and refugee backgrounds. The overarching theme from the interviews was the ability of the CCW Service to act as a ‘bridge to health’ through the provision of culturally responsive care. There were three main categories; supporting access to health and community-based services, improving the healthcare experience, and organisational factors, including part-time hours, capacity, heavy workloads and confusion/lack of clarity regarding the CCW role, which affected CCWs’ capacity to optimally support service providers in providing culturally responsive care. These limitations meant CCWs were not able to meet demand, and fully operationalise the model.

**Conclusion:**

Service providers perceived the CCW model to be a culturally responsive model of care tailored to the needs of women and families from migrant and refugee backgrounds, that reduces barriers to access, and has the potential to improve perinatal outcomes, and women's experience and satisfaction with care.

**Supplementary Information:**

The online version contains supplementary material available at 10.1186/s12905-021-01368-4.

## Background

In 2019, the number of international migrants, individuals living in a country other than their country of birth, was estimated to be almost 272 million globally [1]. A record number of 79.5 million people were forcibly displaced worldwide in 2019 [2]. This includes 26 million refugees who left their country of residence due to fear of persecution, conflict, violence, or human rights violations, and 4.2 million asylum-seekers [2] who have fled their country of residence, sought international protection, but whose refugee status has not been determined [3]. Varied terminology is used to define migrant, refugee, and asylum seeker populations; for the purposes of this study, we used UNHCR definitions [2]. Migrants make a voluntary choice to leave their country of origin and are free to return to their origin country at any time. Conversely, refugees and asylum seekers do not make a voluntary choice to leave their country of origin and are unable to return home in safety [4]. In this study, reference to women from refugee backgrounds included women who are asylum seekers.

Of the total migrant population, 14% are children and 48% are female, of which 59% are of childbearing age [1]. This has significant implications for the planning and delivery of quality maternal, child and family health services in high income countries. Women from migrant and refugee backgrounds who live in high-income countries are at greater risk of adverse perinatal outcomes compared to women born in the host country [5–8]. This includes a higher burden of mental health issues [5, 9–11], pregnancy complications such as preeclampsia, and increased rates of caesarean section [12, 13]. Infants born to migrant mothers are at increased risk of stillbirth [14–17], preterm birth, congenital anomalies [5], and admission to neonatal care units [12, 18, 19]. Women who are refugees or seeking asylum are often particularly vulnerable, due to prior exposure to violence and trauma [20, 21], and are more likely to experience mental health issues, including post-traumatic stress and perinatal depression [22–26].

While migrant and refugee women can often access the same health services as women born in the host country, their challenges may include adapting to a new culture, language barriers, low health literacy, insufficient support to access services, transport issues, and limited financial capacity to pay for care [7, 8, 11, 27, 28]. For many of these women, priorities such as resettlement, housing and finances may be considered more important than pregnancy care [29]. Some migrant and refugee women may also be reluctant to access services due to cultural and social beliefs, and discrimination [30]. They may also experience social isolation, further exacerbating the risk of adverse perinatal outcomes [5, 31, 32]. The health needs of women from migrant and refugee background during the perinatal period is therefore recognised internationally as a public health priority [5, 8, 33–36]. There is a need to develop, implement and evaluate models of care to improve health outcomes, women's experiences and overcome barriers to healthcare access [5, 36–40].

A recent systematic scoping review of pregnancy and postpartum models of care for women from migrant and refugee background living in high income countries (HIC) identified seventeen studies [41]. A diverse range of potentially effective models were identified, including female paraprofessional bicultural/bilingual workers, [42–52], multidisciplinary group models of antenatal care [42, 53], and specialised antenatal clinics [54–56]. All the interventions were acceptable to women; however the review highlighted the need for future research to demonstrate effectiveness, acceptability from the perspective and experiences of women, their partners, family members, and service providers [41].

The Cross-Cultural Workers (CCWs) in Maternity and Child and Family Health Services (the CCW Service) was implemented during 2017 in an area of metropolitan Sydney, Australia. It aims to support women and families from migrant, refugee, and asylum seeker backgrounds to access maternity, child and family health services, and community-based organisations across pregnancy, and through the transition to child and family health services until school entry (5 years).

Within a broader evaluation of effectiveness and acceptability of the CCW Service, this study analyses and describes service providers perceived effectiveness, satisfaction, and recommendations for improvement of the CCW Service.

## Methods

### Design

A sequential explanatory mixed-methods [57, 58] study design using surveys and semi-structured interviews was conducted 18 months after the CCW Service was implemented. A mixed-methods approach is well established in health services research [59, 60], and was chosen for its potential to add rigour, strengthen the significance of results, and allow deeper exploration of service providers experiences and perceptions [61] of the CCW Service. Mixed methods research provides a platform to combine the strengths of both quantitative and qualitative methodologies to offset their respective weaknesses [62]. The sequential explanatory design began with surveys to collect quantitative data followed by semi-structured interviews to collect qualitative data.

### Setting

The CCW Service is based in South-Eastern Sydney, Australia, a culturally diverse metropolitan area. There are three public maternity facilities with approximately 10,000 births per year total, with 38% of women being born in a non-English speaking country [63]. Public antenatal care is offered through antenatal midwifery-led and doctor-led clinics, midwifery group practice, group models of antenatal care, or shared care arrangements between a general practitioner (family doctor) and hospital antenatal clinics. Following birth and discharge home, women and families have access to universal child and family health nursing services provided from birth until school entry.

### The Cross Cultural Worker Service model of care

The CCW Service is an enhanced model of care provided alongside existing maternity and child and family health services. The CCW Service employs three female, part-time, Cross-Cultural Workers (CCWs) who have lived experience of the migration journey, and are fluent in both their country-of-origin language and English. This includes CCWs from Nepal (Nepali and Hindi speaking), Bangladesh (Bangla, Hindi and Urdu speaking) and Indonesia (Bahasa speaking). They focus on supporting women and families from migrant and refugee backgrounds who are socially isolated, financially disadvantaged, have limited support and/or psychosocial risk factors. Although CCW backgrounds do not cover all main origin countries in South-Eastern Sydney (China, India, Pakistan, Thailand, Brazil, and Mongolia are other major origin countries), a 2017 internal perinatal data review and needs analysis report (unpublished) identified Nepali, Bangladeshi, Indonesian and Indian women as being at highest need of additional support and hence the focus of the CCW Service.

CCWs work as part of the multidisciplinary team with midwives, child and family health nurses (CFHN), doctors, allied health and other service providers to: (a) support women and families to navigate maternity, child and family health, and community-based services, (b) enable early access and ongoing service engagement across the continuum of pregnancy and the transition to child and family health services, (c) provide culturally appropriate support to women and their families, defined as care that takes cultural preferences into account [64] and aligned with World Health Organization’s (WHO) standards for improving quality of maternal and newborn care [65, 66]. Such care includes language specific information (when available), CCW with a shared cultural/linguistic background with service users, CCWs acting as cultural brokers or mediators between women and service providers, practical support, and education of both service providers and women, and (d) client advocacy and collaboration with health services, local communities and agencies to build capacity to provide culturally responsive services. The CCWs do not replace accredited interpreters.

The CCWs are co-located in hospitals and community-based clinics. Referrals are received from midwives, doctors, CFHN, allied health and non-governmental organisations (NGO). The CCWs liaise and collaborate with existing service providers to best respond to the client’s individual needs. All client contact occurs during office hours. The number, frequency and type of interaction (including face-to-face, telephone, text messaging, and email contact) varies depending on the preference and needs of each woman and family. The number of contacts can vary from one to ten or more in pregnancy through to school entry. The duration of contact also varies, including a five-minute telephone call, a 30–60-min clinic or home visit, or 1–2-h group education session cofacilitated with clinician/s or NGO worker/s.

Managerial support and clinical governance are provided through a regular meeting with their direct line manager (author and CCW Service Manager HJR), antenatal clinic midwifery manager, and child and family health nursing manager. This provides an opportunity to discuss issues regarding individual clients, workload, client advocacy professional development and boundaries. The CCWs also receive monthly and ad hoc clinical peer supervision from an independent clinical supervisor.

### Study recruitment

Purposive and snowball sampling were used to identify service providers working with women and families from migrant and refugee backgrounds in the study area. Participants were recruited through maternity facilities, child and family health services, NGO and government organisations. An email invitation with details of the purpose of the study was distributed by the two lead researchers (HJR and AH). The email invitation explained the aim of the survey was to hear service providers opinions, views and experience of the CCW Service, and invited the email recipient to participate. The email included a link to the anonymous/no identifying details electronic SurveyMonkey© survey version. Paper copies were also available in service provider workplaces. Completion of the anonymous survey was taken as consent to participate.

The aim was to recruit a minimum 50 participants to complete the survey in order to be confident of inclusion of major groups of maternity and early childhood service providers.

Those who completed the survey were invited to express interest in participating in a semi-structured interview. Purposive sampling via email invitation was also undertaken to ensure representation of service providers who provide client referrals, joint client appointments, support and supervision of the CCW Service. In addition to inviting service providers who were *users* of the CCW Service to participate, the CCWs as *providers* of the Service were also invited to participate in the interviews. This was done because the authors felt their perceptions were uniquely important in presenting the first-hand experiences of role deliverance and capacity to fulfil the Service aims, and such perspectives have been very limited or non-existent in prior related research [41]. The aim was to interview 15–20 service providers, pending when data saturation had been reached.

### Data collection

The survey was developed by study researchers (HJR, AH and CH) based on a literature search of health service evaluation by service providers as well as clinical and research experience, (see Additional file 1) pilot tested by three clinical colleagues, and edited based on feedback. The survey included questions related to participant role and place of work, understanding of the CCW Service, experience with referral, satisfaction with CCW Service integration in maternity, child and family health, and community-based services, achievements, suggestions for improvement, and perceptions of satisfaction and improved care for women. Distribution occurred March–May 2019, with three email reminders sent during this time (balancing sufficient reminders/opportunity to participate against burden to service providers of ongoing emails). The exact email distribution is difficult to estimate as service providers were encouraged to send to colleagues, and Service Managers to their staff, estimated to be approximately 450 people.

Semi-structured face-to-face interviews were conducted June–August 2019. The interviewees included the three CCWs, two participants who provided an expression of interest via survey response, and 14 of the 16 service providers who were suggested by HJR and AH.

An interview guide was initially developed (by HJR, AH and CH) based on a literature search of health service evaluation by service providers, as well as clinical and research experience, (see Additional file 2) pilot tested by LH and DC and revised based on feedback. The survey results from service providers were then also used to inform the final interview guide. Broad open-ended questions were used to elucidate the perspectives and experiences of service providers and to identify key issues. In addition, interview prompts and focused questions were used to clarify issues raised by participants.

All interviews were conducted by LH (student interviewer trained and supervised by DC), rather than the lead researchers (HJR and AH) who had pre-existing professional relationships with interviewees that could influence their responses. Consequently, to ensure confidentiality and anonymity of interview data, identifying information was removed as much as possible from the interviews to limit linking interview responses to individual participants. LH also independently analysed interview data under the supervision of DC to avoid potential influence.

All interview participants were given detailed study information (including regarding anonymity/de-identification of interviews) and gave written informed consent prior to participation. All interviews were digitally recorded, conducted in English, and lasted approximately 25 min (range 8–44 min). All reflections were documented as field notes and used in the analysis. Data saturation was reached after 17 interviews, with no new themes raised in the subsequent two interviews.

### Data analysis

Surveys were analysed using IBM SPSS v.25® (IBM Corporation, Armonk, NY), using descriptive analysis with number (percentage) responses Additionally, free text responses were grouped and counted. Survey data was used to develop the final interview questions that would explore the issues further. All interviews were transcribed verbatim, de-identified and analysed by LH before being cross checked by DC. DC reviewed the coding against the transcripts, and made suggestions to improve the coding structure. The quantitative results were also used as a guide for the coding. A qualitative inductive content analysis approach [67, 68] was used to analyse interview data. Qualitative content analysis involves the researcher identifying, coding and categorising the primary patterns that emerge from the collected data[69, 70].

In the first phase of the qualitative analysis, transcripts were read through repeatedly to obtain an overall perspective. In the second phase, sentences or phrases that contain information that was relevant to the questions were selected. The third phase was a systematic analysis of interview responses. Each interview was read several times and codes that were relevant to the study’s purpose were identified manually by LH using NVivo (Version 12, QSR International Pty Ltd). The multiple codes were sorted into subcategories. These were then merged into main themes after a thorough, comparative analysis [68]. Quotes representative of the coded themes are reported. Quantitative survey data and qualitative interview data were triangulated after the initial analysis phase. Findings were used in a complementary fashion. Initially the survey data were explored, including themes from the free-text sections of the survey. These were then compared with the interview data looking for similarities and differences, in particular, identifying aspects to improve future policy and practice [71].

The study was approved by the South Eastern Sydney Local Health District Human Research Ethics Committee (Approval number: HREC 17/257).

## Results

In total, 69 survey responses were received, and 19 interviews were conducted.

### Survey results

The majority (n = 41, 59%) of respondents were hospital-based, 35% (n = 24) community-based health facility, and 6% (n = 4) community-based NGO. The main professional role of respondents was midwives (n = 26, 37%), and CFHN (n = 21, 30%) (Table 1).

**Table 1:** Professional role of survey participants

Professional role	Number (%)
Midwife	26 (37%)
Child and Family Health Nurse^a^ (CFHN)	21(30%)
Doctor (Obstetrician/Paediatrician)	6 (9%)
Allied Health (Social Worker, Speech Therapist, Health Worker)	6 (9%)
Community Worker with Non-governmental organisation^b^	4 (6%)
Nurse	2 (3%)
Managers	2 (3%)
Administration	2 (3%)
Total	69 (100%)

^a^Child and Family Health Nurses: registered nurse with postgraduate qualifications in child and family health nursing

^b^Non-governmental organization (NGO): Non-profit, voluntary citizens' group that operates independently of government

Most respondents had a good understanding of the CCW Service (Table 2); including the provision of culturally appropriate support (n = 65, 94%), linking clients to supports and networks (n = 63, 91%), supporting engagement with services (n = 60, 87%), access to health information (n = 59, 85%), and culturally responsive service provision (n = 56, 83%). There was some misunderstanding that the CCWs were interpreters (n = 25, 36%) and provided transport (n = 7, 10%).

**Table 2:** Service providers perceptions of what the Cross Cultural Worker Service provides

Role	Number (%)
Culturally appropriate support to women and their families	65 (94%)
Link clients with local community supports and networks	63 (91%)
Support clients to remain engaged with services	60 (87%)
Access to health information	59 (85%)
Supports services to be culturally responsive	57 (82%)
Language specific health information	56 (81%)
Support clients to navigate health and community-based services	54 (78%)
Education, pregnancy and parenting programs	49 (71%)
Support for clients to attend appointments when referred to other services	41 (59%)
Assist clients to attend appointments	33 (48%)
Interpreter service	25 (36%)
Transport	7 (10%)
Other^a^	4 (6%)

^a^Support for psychosocial concerns and working with health professionals to develop culturally appropriate screening processes, information and insight in the area of intellectual disability/autism, not an interpreter service but do translate health information for clients as required, or not sure

Most respondents (n = 44, 64%) had referred to the CCW Service at least once, via email (n = 33, 73%), in person (n = 23, 52%), or by telephone (n = 10, 22%). Overall, 84% of referrers were satisfied with the ease of referral. Almost three-quarters (71%, n = 49) reported that the CCW Service improved integration with maternity services, however less than half (42%, n = 37) said it improved child and family health service integration with 29% (n = 20) unable to say. Almost two-thirds (64%, n = 34) said it improved integration with community-based services, and 68% (n = 46) perceived that women were satisfied with the CCW Service. Table 3 shows service provider perceptions of Service effectiveness, with most (83%) feeling it improved care for women, improved outcomes (68%), and facilitated engagement with the target communities and services (70%); albeit with a high proportion of neutral/unsure regarding outcome improvement (22%) and engagement (17%).

**Table 3:** Service providers perceptions of effectiveness of CCW Service

Service effectiveness	Not effective	Neutral	Effective	Not applicable	Total
Improved care for women	5 (7%)	6 (9%)	57 (83%)	1 (1%)	69 (100%)
Improved outcomes for women	5 (7%)	15 (22%)	47 (68%)	2 (3%)	69 (100%)
Facilitated engagement between target communities and services	6 (9%)	12 (17%)	48 (70%)	3 (4%)	69 (100%)
Collaboration with agencies in health promotion and community development initiatives	6 (9%)	14 (20%)	42 (61%)	7(10%)	69 (100%)

The survey also sought free text responses relating to CCW Service achievements and recommendations for improvement. Achievements included the provision of health education and health promotion for participating women (n = 14), increased access to health and community services (n = 14), supportive and trusting relationship formed with women (n = 14), supports the transition from maternity to child and family health services (n = 4), CCWs employed in the role are a strength of the service (n = 3), and builds capacity of staff to provide culturally responsive services (n = 2). Recommendations for CCW Service (n = 44) improvement included; increase CCW hours or number of workers in the role (n = 16), increase service provider awareness of CCW role and services for women (n = 13), build capacity of service providers to provide culturally responsive services (n = 5), and increase availability of translated information (n = 4).

### Interview results

The 19 interviewees comprised the 3 CCWs and 16 other service providers: 6 midwives (1 at managerial level), 4 child and family health nurses (CFHN), 3 doctors (obstetrics and gynaecology specialists/trainees), 2 NGO workers, and 1 women’s health nurse. 

**Fig. 1:**
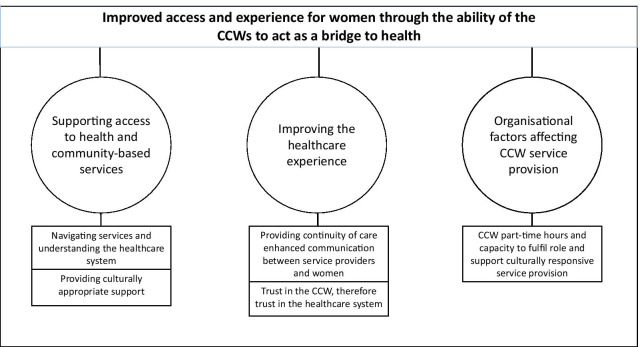
The theme, categories and subcategories from the interviews with service providers

Analysis identified three categories and five subcategories. The overarching theme was improved access and experience of care for women and families through the ability of the CCWs to act as “a bridge to health” through the provision of culturally responsive care. Figure 1 summarises the theme, categories, and subcategories.

### Supporting access to health and community-based services

The CCW Service was seen as being pivotal in supporting access for women and families. The CCWs were well placed and able to refer women and families from migrant and refugee backgrounds to appropriate health and community-based services, and act as a *bridge to health*. The CCWs were able to assist with navigating services and supporting families in understanding the healthcare system, and providing culturally appropriate support.

#### Navigating services and understanding the healthcare system

CCW support was important to assist with accessing and navigating health services. The CCW role was viewed as extending outside health services into the community to link migrant and refugee women with appropriate community-based services, other women, and to support women in their transition to life in Australia. Service providers expressed this by saying:

*It's that absolute bridge and connection in a very meaningful way. And the fact that they feel that they have somebody that they can contact at any time to help them navigate the system but it is about connecting really well with women.* (Midwife6)

*Just to help these women who are usually all alone - they've come from a foreign country usually just with their husbands/partners. Navigating the health system is hard enough just to a person who's lived here… So, they (CCWs) take these women individually basically by the hand and navigate the health system.* (Midwife2)

The CCWs also helped their clients understand how to use the health system, which was seen as critical in ensuring women were able to access care independently. For example:

*I think they're probably more aware of how the system works and have a greater grasp on I guess what their expected interactions are with our clinics and a better overall knowledge of how the hospital works, but also their own health and what we can provide for them medically.* (Doctor2)

Conversely, all three CCWs spoke of women’s reliance on them as a source of “*all information”*. They were very aware of their non-clinician boundaries as facilitators, navigators and information givers only. However, the CCWs described requests for immigration support, where they found it challenging to explain this to the women and families. The CCWs also spoke about service providers initial confusion with the boundaries of their role and being mistaken as a case-worker or interpreter*.* However, the CCWs noted that there had been significant improvements in understanding since they initially commenced in role.

#### Providing culturally appropriate support

The CCW role was perceived to be able to provide culturally appropriate support as they are bilingual, bicultural, and have insights into how the woman is feeling due to their own cultural practices and beliefs, and lived experience of the migration and settlement journey. One midwife described this:

*The CCW can bridge that gap… they have knowledge of the culture and traditions so we can see what those traditions are and then unpack them in terms of whether they are safe or not safe.* (Midwife4)

Conversely, two service providers reported that one CCW for multiple cultures was viewed to be less effective than a CCW focused on a specific culture. One CFHN said:

*I feel that that one culture doesn't necessarily mean that she understands all other cultures or maybe has more of a connection with another culture than any other worker would. The concept of a CCW maybe doesn't work across all cultures.* (CFHN4)

CCW responses largely complemented those from broader service providers. All three CCWs described their role in supporting access and navigation to health and community-based services, improving the healthcare experience through the supportive and close relationship they formed with women, and their ability to understand cultural nuances and reflect this in care. They felt this enhanced the potential for a positive experience of settlement, and promotes social networking with other women and families. The CCWs also recognised the unique role their position inhabits, where they are advocates for the needs of women, families and their community, and providing health services that improve health outcomes. This was explained by one CCW by:

*We provide information during the pregnancy and into transition into parenting and also link them to playgroup, community support or and also to provide any culturally appropriate information or education related to pregnancy and parenting. … advocating for them on behalf of them.* (CCW)

### Improving the healthcare experience

The second theme was about the capacity of the CCW to improve the healthcare experience. Service providers frequently used phrases such as *excellent, successful project, exceptional* and *I don’t ever want them to go*. They felt the CCW Service improved the healthcare experience by making care more personable and less frightening, which supported ongoing service engagement. The key sub-themes were in relation to communication, continuity of care and acknowledging the trusting relationships that women formed with the CCWs.

#### Providing continuity of care enhanced communication between service providers and women

Service providers described continuity of care (often lacking in usual service provision) provided by the CCWs as a key strength. Having one person who women can form a relationship with, and access when they need, enables them to feel more supported and confident, as described by:

*I think they get access to the information that they need and I think that they have somebody that they can call. Because some people don't have anybody that gives them continuity; they have a different midwife every time they come in and I think she provides that role as being the one person you call when you’ve got a problem. And she can take them to the right areas.* (Midwife3)

Enhanced communication between service providers and women was also described as being facilitated by the CCWs as they speak the language, understand the culture, and have a close relationship with the women. Two participants explained:

*Sometimes it's really hard for me as the facilitator just to get them talking because they're all very shy. But I think having the CCW2 and CCW3 there… helps because they can start a conversation […] She (CCW) can start talking about what happened when she was having babies...or maybe say in their language, what I'm trying to get at.* (Midwife2)

#### Trust in the CCW, therefore trusting the healthcare system

Service providers commented that due to the close relationship and supportive role of CCWs with the women, there is a greater level of trust in healthcare system. They felt women were able to access more services because they have formed a trusting relationship with the CCW and have a link with the healthcare system that is positive. For instance:

*I think a little bit of it is definitely that sense of trust. I guess that comes from: “hey this is something I’m (CCW) recommending to you”. Like you know these people can help and support you, and then they come along … we will call an interpreter and … get help if they need it.* (CFHN4)

Trust between women and the CCWs also allowed for cultural norms and practices to be explored and this trusting relationship enabled women to talk about ‘taboo’ subjects such as domestic violence, mental, sexual and reproductive health. For example:

*In the domestic violence space for example a lot of people won't disclose unless it's to a trusted person. It's a really difficult conversation to have anyway, but if you have to do it outside your own language and outside people who you know, who don't understand your culture… Then without that cultural competency and way of knowing how to work with people you know it's not* going to be very effective. (NGO Community Worker1)

Ultimately, service providers explained that by introducing health services and health concepts through a safe and trusted pathway, women from migrant and refugee backgrounds they felt that women feel more empowered to take control of their health at a crucial time in their lives. One doctor said:

*I definitely find that my experience with the women who are labouring who have been part of the groups, they seem to be more educated about their birth and what to expect... I’m meeting them for the first time at three o'clock in the morning, and people that haven't been part of any antenatal education … struggle a lot to understand their state, what their body is going through. So, I think they (CCWs) help facilitate that kind of empowerment for the women.* (Doctor1)

The formation of a trusting relationship between women and the CCWs posed the challenge of CCWs maintaining professional boundaries, especially when working in small communities. They reported that often women expected them to accept tokens of friendship, invitations to join social networks and family events, which initially the CCWs found an ethical dilemma. However, they reported clinical supervision sessions and management support enabled them to develop strategies to manage these situations in a polite and respectful way. Two of the CCWs provided examples of this:

*You cannot in a small community…strictly separate professional from personal… and I've had to politely decline with the fear that they'll be offended if you don't go*. (CCW)

Sometimes when the client says "you and me have not only the professional relationship, but beyond that you know. So why don't you come to my home?" It's a bit difficult and the community is very small, everyone knows each other. (CCW)

### Organisational factors affecting CCW Service provision

The final theme relates to factors which impacted on CCW Service provision. The key sub-theme was the CCW part-time hours and capacity to fulfil role, including, supporting capacity building of service providers to provide culturally responsive care, and governance structures.

#### CCW part-time hours and capacity to fulfil role and support culturally responsive service provision

The biggest challenge reported by service providers was that the part-time hours made it difficult for the CCWs to fulfil their role. Participants who struggled to clearly define the CCWs role called for more “*regular connection”, “in-service education”* or *“better definition of the role.”* Service providers wanted increased numbers of CCWs, increased hours, or the role to become full-time. They felt this would enhance the ability of CCWs to fulfil their role, provide more one-on-one or joint consultations with service providers, and more regular CCW engagement with service providers to raise awareness of the role. One midwife noted:

*They (CCWs) only both work two days a week at the moment. I would say there's enough work for four days a week each…So, it's looking at how much work is there at the moment and how much more they could do if they had more time.* (Midwife4)

The CFHNs in particular described the CCW Service as “*scratching the surface’.* Service providers also expressed concern for the CCWs recognising there is a great potential for CCW ‘*burnout*’ as they are in very high demand.

Some service providers highlighted that it would be beneficial to have the CCWs providing more one-on-one with clients, or involved in service providers consultations. All doctors wished for the CCWs to be more available in antenatal services, in contrast, CFHNs wanted the CCWs in the postnatal period. For instance:

*I'd say almost the majority of our consultations are with women who are from non-English speaking backgrounds or who are from a different culture. So, we're limited in that we don't have the workers there the time that we need.* (Doctor2)

The CCWs ability to fulfill their role was also perceived by service providers to be compromised by the requirement of CCWs to attend meetings, reducing their capacity to reach all women who need the Service. For example:

*Well, I believe she's in a lot of meetings and I don't think you can have one person doing a role that covers a huge area, with a huge number of families, and then go: well, you need to be in meetings… I just think from a managerial point of view, I don't know how you would expect anyone to fill a job to their capacity, yet be in meetings.* (CFHN3)

Similar to service providers, all three CCWs spoke of part-time hours reducing the capacity to fulfil their role and support capacity building of service providers. They described often feeling overwhelmed, and overloaded with their current workload. The CCWs spoke about the need to spread services more equally between antenatal and postnatal services, and one agreed with service providers regarding the need for more regular engagement with staff to describe their role and reiterate what the service can provide for women and families. A key CCW Service strength highlighted by all CCWs was the support they received from their managers, colleagues and peers.

Service providers described how the CCWs built the capacity of their colleagues by raising awareness of the CCW Service as a referral pathway that encourages and supports women to access health services. Additionally, service providers felt that the CCWs were best placed to build capacity to ensure that care is provided in a culturally sensitive way, however perceived this to be limited due to part-time hours. Service providers emphasised the need for more regular engagement with staff to ensure culturally responsive concepts were translated to day-to-day care. For example:

*If the CCWs had sessions with us, the primary care providers on the coal face, because I guess my understanding comes from mostly working with Indigenous women and the training that you get around interpersonal communication, body language, what they (women) appreciate that's culturally appropriate. I haven't been given any of that information … So maybe CCWs actually spending time educating the medical, midwifery staff, capacity building.* (Doctor1)

## Discussion

This study provides insight into the perspectives of service providers regarding the CCW Service for women and families from migrant and refugee backgrounds during pregnancy through to the early parenting period. The CCW Service was highly regarded by service providers and perceived as supporting access to health and community-based services, and improving the healthcare experience for women through the ability of the CCWs to act as a bridge to health. The close supportive and trusting relationship clients formed with the CCWs was perceived to support service navigation, enhance communication between service providers and women, enable continuity of care, and culturally appropriate support, which resulted in trust in the healthcare system and empowered women to consider their health needs. Service providers also perceived the CCW Service to be successful in effectively integrating existing maternity and child and family health services, and supporting women and families in the transition between services.

In contrast, the survey results highlighted that although 70% of service providers perceived engagement between target communities and services to be effective, 9% felt it was not effective and 17% were neutral/unsure. This may genuinely reflect limited or no impact of the Service on targeted community engagement, for example due to part-time CCW hours or the model of care being too recent to have a perceived or actual impact. Additionally, uncertainty may be related to limitations of the perspectives of the service providers using the Service, with some not having knowledge or experience working closely with target communities, and so limited insight into whether the CCW Service was facilitating engagement between target communities and services or not. Similarly, the same reasons may apply to collaboration with agencies in health promotion and community development initiatives, whereby 61% of service providers perceived this to be effective, however 9% felt it was not effective and 20% held a neutral view. Additionally, service providers perception of improved outcomes for women may also be too early to ascertain and requires perinatal health outcome data to support, hence our ongoing research will examine impact on perinatal health outcomes.

A major perceived limitation of the CCW Service was the part-time hours, which did not accommodate the workload or the ability to achieve all the aims of the model of care, namely building the capacity of service providers to provide culturally responsive services. Capacity building also ensures sustainability of culturally responsive service provision for women and families. There was a need to increase working hours, or CCWs in the role to enable further development.

Whilst most service providers were able to clearly define the CCWs role, there was confusion about the scope of the service for some. CCW Service promotion is essential to ensure understanding of CCW roles and responsibilities, capacity, and appropriate referral. Increasing CCW Service visibility is required to dispel role misconceptions, specifically misperception of CCWs as interpreters or case-workers, and better define limitations e.g., part-time hours limits CCW ability to participate in consultations with clinicians, even though theoretically this falls within the scope of their role.

The CCWs reported ethical dilemmas in relation to women’s reliance on them as a source of *all knowledge* and the challenge of maintaining a professional relationship with women while being part of their community. However, they developed strategies to maintain professionalism, without diminishing the trusting relationship developed with women. The CCWs awareness of their role as facilitators and navigators, required explanation to women, that in order to receive the best advice and expertise, referral to clinicians and specialised services, such as immigration was essential. A recent systematic review of models of care in this field found four studies evaluating service provider perspectives [45, 48, 55, 71]. Of these, two explored the perspectives bicultural/bilingual workers [45, 48] employed to support migrant and refugee women and families. Their key roles were to establish a trusting relationship, provide cultural, social, emotional and practical support, assistance to navigate systems, translation, and provision of education and resources [45, 48, 55, 71]. Their ability to enhance communication between women and service providers was perceived to be through lived experience and providing cultural safety [42, 45, 47, 48]. In comparison, our study findings from 69 surveys and 19 interviews with service providers, found the CCWs lived experience of the migration and settlement journey, shared language and cultural understanding enables them to provide culturally appropriate care and support women and families transition to their new life in Australia. This was perceived as critical to ensure ongoing engagement and confidence to access services independently. Additionally, enhanced communication between women and service providers was also integral to the model of care.

Previous studies of bicultural/bilingual workers report their role as core component of the multidisciplinary team in supporting women and families to navigate health systems, providing culturally responsive care, continuity of care, and in-language social, practical and emotional support was highly valued [45, 48]. These findings are consistent with our study, whereby the CCWs were highly regarded for enabling service navigation, culturally responsive care, continuity of care, and psychosocial support.

In our study, the provision of culturally  responsive care was perceived to be enabled by the CCWs sharing lived experience of the migration and settlement journey, and the same language and cultural background as some women. However, not all women and families accessing the CCW Service share the same cultural and language background. Consequently, two service providers reported that one CCW for multiple cultures was less effective than a CCW focused on a specific culture, as without common language and cultural understanding the CCW couldn’t really provide more support and services that than a nurse or midwife themselves. Thus, transferability and the implementation of similar models of care requires individual consideration to the cultural and language background of women accessing services, and the concept of working across multiple cultures or matching the CCW to a specific cultural or language group. Additionally, consideration to lived experience of the migration and settlement journey being an important component in supporting women and families from multiples cultures. Continuity of carer was also identified as a critical component to improve care, enhance service access, reduced the need for women to revisit traumatic memories, and enabled effective communication in conversations surrounding culturally ‘taboo’ subjects [55]. Similarly, in our study, service providers highlighted the CCWs role in developing a trusting relationship, providing continuity and culturally responsive care enhanced communication and discussion of taboo topics.

Maintaining the balance of client workload, advocacy, clinical supervision, professional development, and governance is challenging, especially when the CCWs work part-time hours. There is also the demand for cultural expertise, co-design, community engagement and collaborative projects with key stakeholders, which is limited by current part-time hours. Consequently, clinical supervision, effective management and support are imperative to this model of care.

Overall, service providers reported improved integration with maternity and child and family health services. However, some service providers reported there was more of a focus on maternity compared to child and family health services. The CCWs noted that due to part-time hours there was a need to prioritise demand, and often pregnancy seemed the time of greatest need. Working across the continuum of pregnancy and the transition to early parenting, whilst maintaining a balance between maternity and child and family health services is as a key challenge. One mechanism to manage this challenge is regular joint meetings that involve the CCWs, maternity and child and family health managers to discuss issues and ensure shared decision making to resolve issues as they arise.

## Strengths and limitations of the study

A strength of this study is its focus on the understudied area of models of perinatal care for women and families from migrant and refugee backgrounds, specifically from the perspective of service providers. A mixed-methods design was chosen to allow deeper exploration of service providers perceptions, and strengthen the significance of results. Additionally, we captured the rarely explored perspective of the CCWs themselves. Their perspective is uniquely important as it presents the first-hand experiences of service provision and capacity to fulfil the Service aims.

Limitations include that we were unable to accurately ascertain the survey response rate due to snowballing distribution by the study researchers, Service Managers and colleagues. We estimate approximately 450 staff were invited, a low response rate of 15%. However, many of these staff would have minimal interaction with the CCWs (e.g., those working in Birth Services only), so would not have seen it as directly relevant and be unlikely to complete it. Hence the response rate of relevant staff is likely higher. The survey also included an invitation to participate in an interview, with only two volunteers. Consequently, service providers were suggested by the study researchers to ensure representation of all the major stakeholders. The small sample size and model of care limit the generalisability of this study. However, it does allow us to understand, from the perspective of service providers, and the CCWs, the strengths and challenges of such as initiative in the Australian healthcare system. Whilst the number of participants who completed surveys and interviews was representative of service providers involved in the CCW Service, i.e., midwives, CFHN, doctors and NGO staff, we acknowledge that these views may not be transferrable to similar groups. However, the findings may be generalised to other countries with similar maternity systems to Australia.

The lead researchers, HJR and AH due to their relationship with study participants, are cognizant that even with the survey being anonymous, and the interview data being deidentified, participants may provide “socially desirable” responses due to their relationship with the researchers. In addition, the main study author and researcher (HJR), is responsible for management of the CCW Service and CCWs, and is a nurse/midwife, hence acknowledges the values, opinions and experiences brought to this study and potential influence on data interpretation. This potential influence has been avoided as much as feasible, by interviews being conducted independently and analysed by LH under the supervision of DC.

Although average length of interviews was 25 min, some were quite brief (range 8–44 min). Therefore, some interviews may have been quite superficial in their exploration of the CCW Service. However, as interviews were performed until data saturation, we are confident that overall, all major relevant themes are likely to have been captured.

Terminology and identification of migrants, refugees and asylum seekers is widely diverse and often inconsistent which pose challenges, including access to health care [4]. Refugee and asylum seeker women experience additional perinatal and mental health risk factors and vulnerabilities when compared to women who are economic migrants. Health research tends not to disaggregate refugees, and asylum seekers from migrant women [24]. This results in literature that obscures the perinatal health, consequently future research and models of care need to specifically address the needs of migrant, refugee and asylum seeker populations individually [4, 23, 24, 69].

Current funding of the CCW Service is limited to the existing hours, however opportunities for additional funding are continuously explored. The findings reported here are part of a larger mixed-methods study of the CCW Service. Our ongoing research will explore effectiveness and acceptability of the Service from the perspective and experiences of women, their partners, and impact on perinatal health outcomes. In turn this has potential to support future funding and scalability, to enable improved care for women and families from migrant and refugee backgrounds.

## Conclusion

In this study of a pregnancy and postnatal model of care for migrant and refugee women in metropolitan Australia, we found that the CCW Service was highly regarded by service providers and seen as integral to the provision of culturally responsive care across the continuum of pregnancy to the transition to child and family health services. Suggestions for improvement included increased hours in order to meet demand, maintain a balance across between maternity and child and family health services, and building service provider capacity to provide culturally responsive care).

## Supplementary Information

**Additional file 1.** Cross Cultural Workers in Maternity and Child and Family Health Services: Survey for service providers. Custom-created survey for the purpose of this study.

**Additional file 2.** Cross Cultural Workers in Maternity and Child and Family Health Services: Semi-structured interview guide for service providers. Custom-created survey for the purpose of this study.

## Data Availability

The datasets used and/or analysed during the current study are not publicly available as service providers were not asked to give consent for the transcripts to be published in their entirety. Datasets are available from the corresponding author on reasonable request. Service providers did consent to their professional status to be disclosed. Quotes in this paper have been selected in a manner which assures the individual source is not identifiable.

## Abbreviations

CCWs: Cross Cultural Workers
the CCW Service: The Cross Cultural Worker Service
CFHN: Child and Family Health Nurse
NGO: Non-Governmental Organisation
HJR: Helen J Rogers
LH: Lily Hogan
DC: Dominiek Coates
AH: Amanda Henry
CH: Caroline Homer
