# Supplementary material for: Cross Cultural Workers for women and families from migrant and refugee backgrounds: a mixed-methods study of service providers perceptions
Source: BMC Womens Health. 2021 May 27;21:222. doi: 10.1186/s12905-021-01368-4 (PMC8161620; doi:10.1186/s12905-021-01368-4)
Supplement: Supplementary file 1 — Additional file 1. Cross Cultural Workers in Maternity and Child and Family Health Services: Survey for service providers. Custom-created survey for the purpose of this study. [file 12905_2021_1368_MOESM1_ESM.pdf]

Additional file 1

Cross Cultural Workers for women and families from migrant and refugee backgrounds: A mixed-methods study of service providers perceptions

Authors' list

Helen J. Rogers PhD Candidate, RM, RN, MPH [1 2]

Lily Hogan Medical Student [2]

Associate Professor Dominiek Coates PhD [3]

Professor Caroline SE Homer RM, MN, MMedSc(ClinEpi), PhD [3 4]

Associate Professor Amanda Henry PhD MPH FRANZCOG BMed BMedSci, DDU (O&G) [2 5 6]

Author affiliations

[1] Child, Youth & Family Services, South Eastern Sydney Local Health District, Sydney, NSW, 2010, Australia

[2] School of Women's and Children's Health, University of NSW (UNSW), Sydney, NSW, 2000, Australia

[3] Centre for Midwifery and Child and Family Health, Faculty of Health, University of Technology Sydney, NSW, 2007, Australia

[4] Maternal and Child Health, Burnet Institute, Melbourne, Vic, 3004, Australia

[5] Department of Women's and Children's Health, St George Hospital, Sydney, NSW, 2217, Australia

[6] Australia Global Women's Health Program, The George Institute for Global Health, Sydney, NSW, 2042, Australia

Cross Cultural Workers in Maternity and Child & Family Health Services - Service Providers

You are invited to participate in research on the Cross Cultural Workers in Maternity and Child & Family Health Services in South Eastern Sydney Local Health District (SESLHD).

The purpose of this research is to hear from you, service providers, about your opinions and experiences of the Cross Cultural Workers (CCW) in Maternity and Child & Family Health Services, and whether it makes it easier for women and families from migrant or refugee background to obtain support, education and information to improve health outcomes for women and their children. The Cross Cultural Worker model of care employs Cross Cultural Workers to:

- Support women and families to access and maintain ongoing engagement with pregnancy and child and family health services
- Link women and families to pregnancy, child and family health services, and community supports
- Provide culturally appropriate information and education to women and their families, including language specific information about pregnancy, parenting and support services.

This survey is one aspect of the research. After completing the survey, you can also volunteer to participate in an interview, to provide more detail about your experiences and views of the CCW Service. This interview would be for approximately 60 minutes and would occur at a time and place convenient for you. Not all individuals who volunteer for an interview will be contacted, as there will only be a small number of interviews. The interviews will be conducted by an independent facilitator.

The decision to be involved in one or both parts of the research is completely voluntary. If you do decide to be involved, your contribution to and experience of this CCW Service is very important to us as it helps us understand the quality of the CCW Service and allows us to see where we need to improve.

Who is carrying out this research?

The research is being conducted by Helen Rogers, Early Parenting Program Coordinator, SESLHD and Coordinating Investigator, under the supervision of Dr Amanda Henry, School of Women's and Children's Health, University of New South Wales (UNSW) Medicine.

About this survey

This survey aims to explore your opinions and experiences of the CCW Service. There are no right or wrong answers. We just want to know your thoughts.

What about confidentiality?

All responses to the survey will be confidential, your name or other information that could identify you will not be used.

Who will read what I write?

Only the research team will have access to your responses.

How long is the survey?

The survey should take approximately 15-20 minutes to complete.
Once you have read through and are comfortable with the information provided, please continue and complete the survey.

What happens after I have completed the survey?

After completing the survey, you can volunteer for an interview, to provide more detail about your experience and views. A summary of the research and its findings will be provided to all service providers who participate in the research.
If you have any questions about this research please contact:
Helen Rogers - Coordinating Investigator

Telephone 02 9382 8685 Email: Helen.Rogers@health.nsw.gov.au

Thank You in advance for taking the time to complete the survey.

* 1. What is your role? Please tick all that apply

- [ ] Midwife
- [ ] Child and Family Health Nurse
- [ ] Nurse
- [ ] Social Worker
- [ ] Community Worker
- [ ] Other Please specify
- [ ] Project Officer
- [ ] Non-Government Organisation Worker
- [ ] General Practitioner
- [ ] Obstetrician
- [ ] Paediatrician

* 2. What hospital or service do you work for?

- [ ] The Royal Hospital for Women
- [ ] St George Hospital
- [ ] The Sutherland Hospital
- [ ] Sydney Children's Hospital
- [ ] Sydney Children's Hospital Community Health Services
- [ ] Other (please specify)
- [ ] St George and Sutherland Child and Family Health Service
- [ ] Non-Government Organisation
- [ ] Central and Eastern Primary Health Network
- [ ] Family Referral Service

* 3. What is your understanding of what the Cross Cultural Workers (CCW) Service is, please describe:

* 4. Have you referred a women or family to the CCW Service?

- ( ) Yes
- ( ) No Please go to question 7

5. If Yes, how did you make the referral? Please tick all that apply

- [ ] Telephone
- [ ] Referral Form and emailed to CCW
- [ ] Referral Form and faxed to CCW
- [ ] Other (please specify)
- [ ] eMR CHOC
- [ ] Email request
- [ ] In person

6. Overall, how satisfied were you with the ease of referral to the CCW Service?

- Very satisfied
- Somewhat satisfied
- Neither satisfied nor dissatisfied
- Somewhat dissatisfied
- Very dissatisfied

Please provide any comments

* 7. From the list below, which do you think the CCW Service provides? Please tick all that apply

- [ ] Assist clients to attend appointments
- [ ] Support for clients to remain engaged with services
- [ ] Support for clients to attend appointments when referred to other services
- [ ] Access to health information
- [ ] Culturally appropriate support to women and their families
- [ ] Language specific health information
- [ ] Interpreter service
- [ ] Transport
- [ ] Education, pregnancy and parenting programs
- [ ] Support with navigating Health, non-government and other government services
- [ ] Links clients with local community supports and networks, e.g. parenting services, playgroups
- [ ] Supports services to be responsive to the needs of women of migrant and refugee backgrounds
- [ ] Other, please specify

8. How satisfied are you with the CCW Service integration with the care that is already provided by Maternity services?

- Very satisfied
- Somewhat satisfied
- Neither satisfied nor dissatisfied
- Somewhat dissatisfied
- Very dissatisfied
- Don’t know/cannot say

Please provide any comments

9. How satisfied are you with the CCW Service integration with the care that is already provided by Child and Family Health Services?

- Very satisfied
- Somewhat satisfied
- Neither satisfied nor dissatisfied
- Somewhat dissatisfied
- Very dissatisfied
- Don’t know/cannot say

Please provide any comments

10. How satisfied are you with the CCW Service integration with the care that is already provided by community based services?

- Very satisfied
- Somewhat satisfied
- Neither satisfied nor dissatisfied
- Somewhat dissatisfied
- Very dissatisfied
- Don’t know/cannot say

Please provide any comments

11. How satisfied do you think women are with the CCW service?

- Very satisfied
- Somewhat satisfied
- Neither satisfied nor dissatisfied
- Somewhat dissatisfied
- Very dissatisfied
- Don’t know/cannot say

Please provide any comments

* 12. Do you think women have received improved care as a result of the CCW service?

1=Not at all. 3=Neutral. 5=A great deal. N/A=Not applicable.

1	2	3	4	5	N/A
○	○	○	○	○	○

Other (please specify)

* 13. Do you think outcomes for women and families have improved as a result of the CCW Service?

1=Not at all. 3=Neutral. 5=A great deal. N/A=Not applicable.

1	2	3	4	5	N/A
○	○	○	○	○	○

Other (please specify)

* 14. How effective do you think the CCW Service has been in facilitating engagement between migrant and refugee communities and maternity, child and family health and community based services?

1=Not at all. 3=Neutral. 5=A great deal. N/A=Not applicable.

1	2	3	4	5	N/A
○	○	○	○	○	○

Please provide any comments

* 15. How effective do you think the CCW Service has been in regard to collaborating with other agencies in health promotion and community development initiatives?

1=Not at all. 3=Neutral. 5=A great deal. N/A=Not applicable.

1	2	3	4	5	N/A
○	○	○	○	○	○

Please provide any comments

16. Can you provide any examples of key achievements of the CCW Service?

17. Do you have any suggestions of how the CCW Service could be improved or enhanced?

18. Do you think there are new and emerging population groups in SESLHD which may indicate the need for a change in focus for the Service?

19. Please provide any additional comments you believe are relevant to the research

Now that you have completed this survey, you can volunteer to be selected to take part in an interview. The aim of the interview is to obtain a greater understanding of your experience of working with Cross Cultural Workers (CCW) in Maternity and Child & Family Health Services.

If you are interested in volunteering to be part of the next part of the research please provide your contact details and best times to make contact. As stated earlier, your privacy will be protected.

NB: The interview will be facilitated by an independent party and not the research team

Link to volunteer to participate in an interview **HERE**

Thank you for completing this survey. Your participation is greatly appreciated.

If you have any concerns or complaints about the conduct of this study, you should contact the Research Support Office of the South Eastern Sydney Local Health District Human Research Ethics Committee which is nominated to receive complaints from research participants. You should contact them on 02 9382 3587, or email SESLHD-RSO@health.nsw.gov.au and quote HREC 17/257.
