# Supplementary material for: Cross Cultural Workers for women and families from migrant and refugee backgrounds: a mixed-methods study of service providers perceptions
Source: BMC Womens Health. 2021 May 27;21:222. doi: 10.1186/s12905-021-01368-4 (PMC8161620; doi:10.1186/s12905-021-01368-4)
Supplement: Supplementary file 2 — Additional file 2. Cross Cultural Workers in Maternity and Child and Family Health Services: Semi-structured interview guide for service providers. Custom-created survey for the purpose of this study. [file 12905_2021_1368_MOESM2_ESM.docx]

**Additional file 2**

**Cross Cultural Workers for women and families from migrant and refugee backgrounds: A mixed-methods study of service providers perceptions**

**Authors' list**

Helen J. Rogers PhD Candidate, RM, RN, MPH ^1 2^

Lily Hogan Medical Student ^2^

Associate Professor Dominiek Coates PhD ^3^

Professor Caroline SE Homer RM, MN, MMedSc(ClinEpi), PhD, ^3 4^

Associate Professor Amanda Henry PhD MPH FRANZCOG BMed BMedSci, DDU (O&G) ^2 5 6^

**Author affiliations**

^1^ Child, Youth & Family Services, South Eastern Sydney Local Health District, Sydney, NSW, 2010, Australia

^2^ School of Women’s and Children’s Health, University of NSW (UNSW), Sydney, NSW, 2000, Australia

^3^ Centre for Midwifery and Child and Family Health, Faculty of Health, University of Technology Sydney, NSW, 2007, Australia

^4^ Maternal and Child Health, Burnet Institute, Melbourne, Vic, 3004, Australia

^5^ Department of Women’s and Children’s Health, St George Hospital, Sydney, NSW, 2217, Australia

^6^ Australia Global Women’s Health Program, The George Institute for Global Health, Sydney, NSW, 2042, Australia

**Cross Cultural Workers in Maternity and Child and Family Health Services: Semi-structured interview guide for service providers.**

- In general terms, please describe your involvement with the Cross Cultural Workers (CCW) in Maternity and Child & Family Health Services
- What do you consider are the main aims and components of the CCW Service?
- If you think there have been, can you please tell me about the strengths and achievements of the CCW Service?
- If you think there are, can you please tell me about any limitations of the CCW Service?
- How effective have the arrangements been for governance, management and reporting for the CCW Service?
- Do you have any suggestions for improving the CCW Service?
- Do you think there are new and emerging population groups in the local area which indicate the need for a change in focus of the CCW Service?
- Is there anything else you would like to add?
